# Impact of frozen elephant trunk on the outcomes of thoracoabdominal aortic repair with normothermic iliac perfusion

**DOI:** 10.3389/fsurg.2022.1044089

**Published:** 2023-01-06

**Authors:** Haoyu Gao, Luchen Wang, Yanxiang Liu, Shenghua Liang, Bowen Zhang, Jie Ren, Cuntao Yu, Xiaogang Sun

**Affiliations:** Department of Cardiovascular Surgery, State Key Laboratory of Cardiovascular Disease, Fuwai Hospital, National Center for Cardiovascular Diseases, Chinese Academy of Medical Sciences and Peking Union Medical College, Beijing, China

**Keywords:** frozen elephant trunk, thoracoabdominal aortic repair, normothermic iliac perfusion, logistic regression, aortic surgery, risk facors

## Abstract

**Background:**

Frozen elephant trunk technique (FET) has been proven to provide an excellent landing zone for second-stage thoracoabdominal (TA) aortic repair. The aim of this study was to evaluate the impact of FET in TA aortic repair with normothermic iliac perfusion.

**Methods:**

From January 2008 to December 2019, 144 patients undergoing TA repair with normothermic iliac perfusion were enrolled in this study. Early and mid-term outcomes of patients with previous FET implantation (group A, *n* = 62) were compared with patients without previous FET implantation (group B, *n* = 82). The logistic regression analysis was performed to investigate the risk factors for adverse events, which were deﬁned as early death, permanent stroke, permanent paraplegia, or permanent renal failure necessitating dialysis.

**Results:**

The proximal aortic clamp time and operating time was 14.26 ± 5.57 min and 357.40 ± 94.51 respectively in group A, which were both significantly shorter than that in group B (18.67 ± 5.24 min and 18.67 ± 5.24 min). The incidence of adverse event was significantly lower in group A than that in group B (9.7% vs. 25.6%, *P* = 0.027). There was no significant difference between two groups with regard to other complications or late outcomes. In addition, age >50 years, a Ccr < 90 ml/min/1.73 m^2^ and the operating time were identified as significant risk factors through logistic regression analysis for adverse events of TA repair.

**Conclusions:**

The FET technique simplifies the operative technique of proximal anastomosis, decreases the operating time and improves the early outcomes in TA repair, whereas does not provide a significant benefit with regard to late outcomes. Long-term follow-up and studies with larger sample sizes are necessary for further confirmation.

## Introduction

Thoracoabdominal aortic (TA) repair is the most common in the management of extensive aortic pathology, but it has substantial morbidity and mortality. Coselli et al. reported the outcomes of 3,309 patients with TA repairs and showed that the morbidity rate was 7.5% and the ten-year survival rate was 36.8%. The Normothermic iliac perfusion strategy was proven to be a viable alternative with preferable outcomes compared with deep hypothermic circulatory arrest (DHCA) (1). However, fragile aortic tissues due to the inflammatory response delayed the proximal anastomosis time and aortic clamp time. At the same time, dissection of the distal aortic arch also has the potential to cause iatrogenic injury to the pulmonary artery, esophagus, and local nerves. In 1983, Borst and colleagues introduced the elephant trunk technique, which facilitated proximal anastomosis in TA repair (2). Subsequently, the frozen elephant trunk (FET) approach was developed in Stanford type A aortic dissection (AAD) or aortic arch disease and was recently considered to treat complicated Stanford type B aortic dissection (3–5). Additionally, this technique provided an excellent landing zone for second-stage TA repair. In this study, we analyzed the early and mid-term outcomes of patients undergoing normothermic TA repair with previous FET implantation or without previous FET implantation to evaluate the effect of FET on TA repair.

## Patients and methods

### Patients

Between January 2008 and December 2019, 144 patients undergoing thoracoabdominal aortic repair with normothermic iliac perfusion according to the European Society for Vascular Surgery guidelines were enrolled in this study (6). All patients underwent preoperative computed tomographic angiography (CTA) of the thoraco-abdominal aorta. The extent of the repair included descending aorta to iliac aorta. The exclusion criteria were as follows: patients who had thoracoabdominal aortic repair under DHCA and patients who had their extent of repair not involving the intercostal artery, celiac axis, superior mesenteric artery (SMA) or renal artery. All patients were divided into two groups according to the previous FET implantation. A risk factor analysis was conducted among all of the patients. Follow-up data were obtained by the outpatient clinic or by telephone consultation. The study protocol was reviewed and approved by the Ethics Committee of Fuwai Hospital (No. 2021-1557), with informed consent obtained to use clinical data in research.

### Study definition

Early death was defined as in-hospital death and 30-day death, including death during operation (7). Complications were defined according to the joint Society of Thoracic Surgeons (STS)-ESTS definitions (8). Paraplegic patients were defined as patients with lower-extremity neurologic deﬁcits due to spinal cord ischemia (SCI) but not stroke. The complications were considered permanent if they were present at the time of hospital discharge or if the patient had an early death. Adverse events, which were the composite end points of the study, included early death, permanent stroke, permanent paraplegia, and permanent renal failure necessitating dialysis. The reoperations were due to excessive bleeding or the failure of the repair involving pseudoaneurysm, ﬁstula, or graft infection, which necessitated surgical exploration or repair. The preoperative creatinine clearance rate (Ccr) was calculated according to the Cockcroft-Gault equations and was classified into five stages: I (≥90 ml/min/1.73 m^2^), II (60–89 ml/min/1.73 m^2^), III (30–59 ml/min/1.73 m^2^), IV (15–29 ml/min/1.73 m^2^), and V (<15 ml/min/1.73 m^2^).

### Surgical strategies

**Figure 1:**
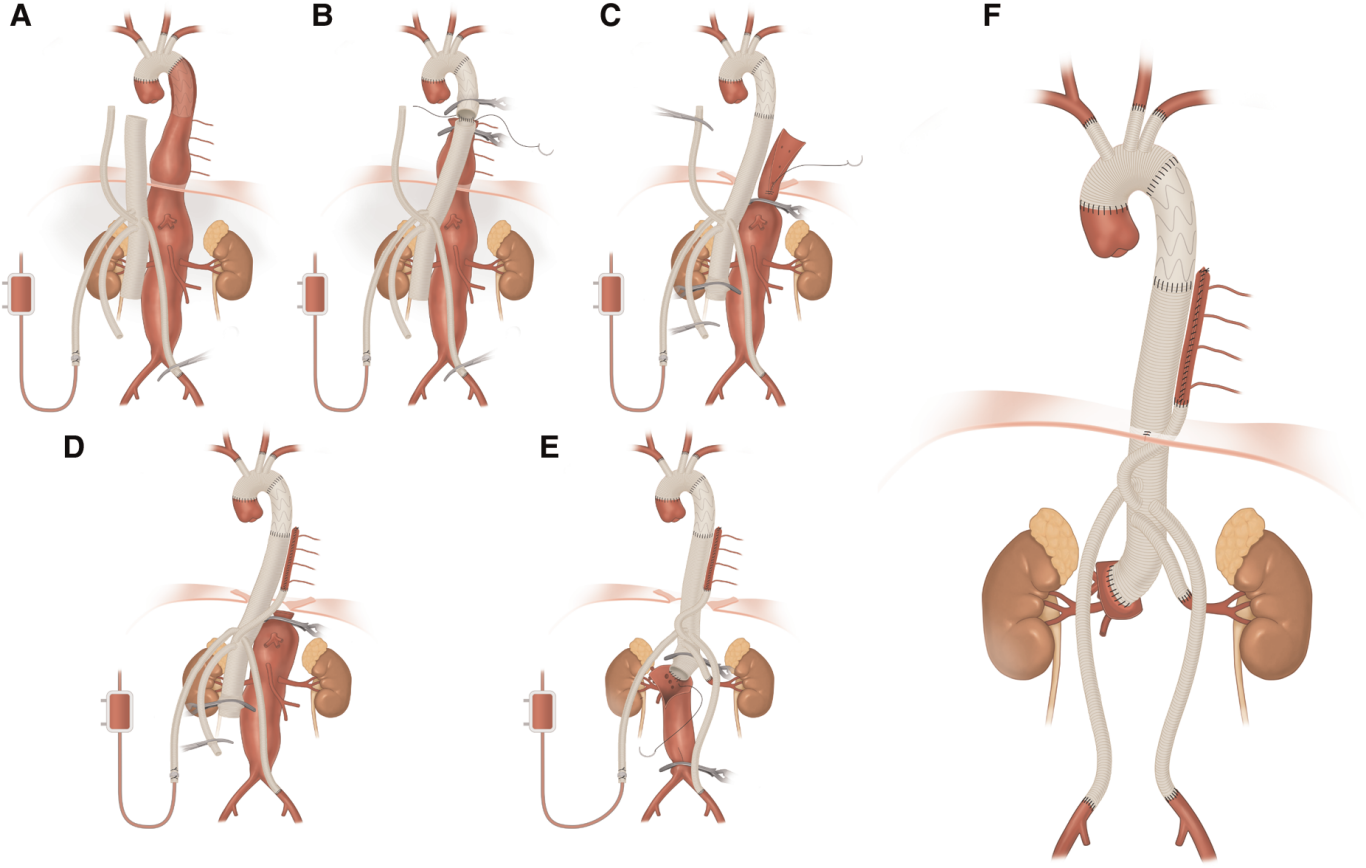
Thoracoabdominal aortic repair with normothermic iliac perfusion. (**A**) Anastomosis of left common iliac artery; (**B**) Proximal anastomosis; (**C,D**) Tube technique to restore the blood ﬂow to the spinal cord; (**E**) Anastomosis of SMA, the celiac axis and the renal artery; (**F**) Anastomosis of right common iliac artery.

All thoracoabdominal aortic repairs were performed under an iliac perfusion strategy with the assistance of a warming blanket to maintain a nascent temperature >35°C. Surgical strategies were shown in Figure 1. We applied a 4-branched graft to replace the diseased thoracoabdominal aorta. First, one branch was anastomosed to the left common iliac artery, and the cannula of the conventional heart lung machine was inserted into the other branch. Blood from the surgical field was retrieved by a right heart aspirator. Then, the retrieved blood was pumped back into the body with the assistance of a conventional heart-lung machine through the iliac cannula. Subsequently, the proximal thoracic aorta or FET was clamped, and the proximal end of the 4-branched graft was anastomosed to the proximal thoracic aorta or FET. Then, one branch of the 4-branched graft was anastomosed with the T8–L2 intercostal or lumbar artery oriﬁces based on the tube technique to restore the blood ﬂow to the spinal cord. After abdominal aortic cross-clamp release, cold crystalloid solution for renal and normothermic blood for the celiac axis was perfused through a urinary catheter to protect visceral function. The origins of 3 arteries (the SMA, the celiac axis and the right renal artery) were anastomosed with the distal end of the 4-branched graft based on the island technique. The origin of the left renal artery was anastomosed with one branch of the 4-branched graft. Finally, the cannula was extracted from the branched graft, which was then anastomosed with the right iliac artery. More details of this technique have been described in a previous study (1).

For proximal anastomosis of patients with previous FET implantation, the routine aortic cross-clamp was placed on the distal FET and descending aorta respectively. Surgeons should be aware of that extensive thrombus usually existed between the FET and the aortic wall in patients with endoleak and needed more time to be cleaned up. Therefore, the dissection of the distal FET from the aortic wall was a key step in the whole procedure of proximal anastomosis. Then, the proximal end of the 4-branched graft was anastomosed to the FET using 4-0 Prolene. After the proximal aorta was unclamped, the teflon felt was selectively used for reinforcement according to the bleeding situation of the anastomosis.

### Statistical analysis

Data were analyzed with R software (version 3.6.1). Count data were expressed as n (%). Continuous variables with a normal distribution are presented as the mean ± standard deviation, and continuous variables with a nonnormal distribution are presented as the median [interquartile range]. The Pearson chi-square test or Fisher exact test was applied for count data. The independent sample t-test was used for normally distributed data, and the Wilcoxon test was used for nonnormally distributed data. A *P*-value <0.05 indicated statistically signiﬁcant differences. The risk factors for adverse events were evaluated. Logistic regression was conducted for single variable and multivariable risk factor analysis. The threshold of the single variable analysis was a *P*-value <0.1, and the threshold for the stepwise multivariable logistic regression was a *P*-value <0.05. Kaplan–Meier analysis was performed using the “survminer” R package, and the cutoff value was *P* < 0.05.

## Results

### Preoperative characteristics

The mean age of all of the patients was 46.5 ± 12.7 years. One hundred and eight patients were male (75%). Sixty-two participants underwent FET implantation before surgery and were divided into group A. The remaining patients who had not previously underwent FET implantation were divided into group B. One hundred and thirty-five patients were diagnosed as aortic dissection, whereas nine patients were diagnosed as aortic aneurysm without dissection. There was no significant difference in the essential patient characteristics, concomitant diseases, or organ function between the two groups (Table 1).

**Table 1:** Patient characteristics.

Variables	Group A (*N* = 62)	Group B (*N* = 82)	*P*-value
Age, *n* (%)			1
<50 years	32 (51.6)	43 (52.4)	
≥50 years	30 (48.4)	39 (47.6)	
Sex, *n* (%)			1
Female	15 (24.2)	21 (25.6)	
Male	47 (75.8)	61 (74.4)	
BMI, *n* (%)			0.607
<18.5 (kg/m^2^)	9 (14.6)	7 (8.5)	
18.5 ≤ BMI < 25 (kg/m^2^)	31 (50.0)	47 (57.3)	
25 ≤ BMI < 30 (kg/m^2^)	18 (29.0)	21 (25.6)	
<30 (kg/m^2^)	4 (6.5)	7 (8.5)	
Aortic dissection, *n* (%)	60 (96.8)	75 (91.5)	0.339
Aortic aneurysm without dissection, *n* (%)	2 (3.2)	7 (8.5)	0.339
Connective tissue disorder, *n* (%)	29 (46.8)	29 (35.4)	0.226
Hypertension, *n* (%)	48 (77.4)	68 (82.9)	0.539
Hyperlipidemia, *n* (%)	21 (33.9)	39 (47.6)	0.139
Diabetes, *n* (%)	0	3 (3.7)	0.351
Coronary artery disease, *n* (%)	6 (9.7)	5 (6.1)	0.628
Stroke (%)	3 (4.8)	9 (11.0)	0.310
Peripheral vascular disease, *n* (%)	5 (8.1)	5 (6.1)	0.898
Maximum distal aortic diameter, cm [mean (SD)]	7.93 (8.63)	8 (8.95)	0.960
Preoperative creatinine, µmol/L [mean (SD)]	89.37 (19.44)	89.30 (23.21)	0.984
Ccr, †*n* (%)			0.108
I	38 (61.3)	38 (46.3)	
II	18 (29.0)	38 (46.3)	
III	6 (9.7)	6 (7.3)	
Preoperative ALT, U/L [mean (SD)]	18.17 (13.10)	16.65 (10.16)	0.432
Preoperative AST, U/L [mean (SD)]	17.42 (7.86)	17.15 (6.47)	0.820
Preoperative amylase, U/L [mean (SD)]	60.89 (65.64)	62.28 (36.22)	0.871
Emergency, *n* (%)	1 (1.6)	5 (6.1)	0.362

Values are *n* (%) or median [interquartile range]. BMI, body mass index; PCI, percutaneous coronary intervention; SMA, superior mesenteric artery; Ccr, creatinine clearance rate; ALT, alanine aminotransferase; AST, aspartate aminotransferase; †Ccr was calculated among creatinine according to Cockcroft-Gault equations, and was classified into five stage, including I (>90 ml/min/1.73 m^2^), II (60–89 ml/min/1.73 m^2^), III(30–59 ml/min/1.73 m^2^), IV (15–29 ml/min/1.73 m^2^), V(<15 ml/min/1.73 m^2^), there was no patients with Ccr IV and V in this study.

### Intraoperative data and early outcomes

The intraoperative data and early outcomes of the two groups are summarized in Tables 2, 3. Notably, the proximal aortic clamp time and operating time was 14.26 ± 5.57 min and 357.40 ± 94.51, respectively in group A, which were both significantly shorter than that in group B. However, there was no significant difference in the spinal cord ischemia time, intraoperative minimum temperature, intraoperative blood loss or blood product use (Table 2).

**Table 2:** Operative details.

Variables	Group A	Group B	*P*-value
Proximal aortic clamp time, min [mean (SD)]	14.26 (5.57)	18.67 (5.24)	<0.001
Time of spinal cord ischemia, min [mean (SD)]	12.92 (5.32)	13.74 (4.71)	0.342
Operating time, min [mean (SD)]	357.40 (94.51)	402.13 (117.45)	0.015
Minimum temperature, [mean (SD)]	35.23 (0.81)	35.26 (0.63)	0.793
Blood loss, ml [median (IQR)]	1050.00 [900.00, 1400.00]	1140.00 [900.00, 1750.00]	0.482
Transfusion requirements			
Packed red blood cells, units [median (IQR)]	4.00 [2.00, 5.50]	3.50 [2.00, 5.50]	0.396
Fresh frozen plasma, ml [median (IQR)]	400.00 [0.00, 800.00]	600.00 [0.00, 800.00]	0.825
Platelet, units [median (IQR)]	2.00 [2.00, 2.00]	2.00 [2.00, 3.00]	0.777

Values are *n* (%), mean (standard deviation), or median [interquartile range].

**Table 3:** Early outcomes.

	Group A	Group B	*P*-value
Intubation time, h [mean (SD)]	54.97 (93.98)	51.91 (72.17)	0.826
Length of ICU stay, h [mean (SD)]	152.52 (136.76)	157.24 (191.40)	0.869
Hospital stay, d [mean (SD)]	16.74 (7.94)	16.52 (10.68)	0.893
POD1 drainage, ml [mean (SD)]	668.87 (359.19)	739.51 (1035.64)	0.608
Drainage time, d [mean (SD)]	11.56 (4.96)	10.87 (7.92)	0.543
Adverse event, *n* (%)	6 (9.7)	21 (25.6)	0.027
Early death, *n* (%)	3 (4.8)	9 (11.0)	0.31
Intraoperative death, *n* (%)	0 (0.0)	1 (1.2)	1
In-hospital death, *n* (%)	1 (1.6)	6 (7.3)	0.236
30 days death, *n* (%)	2 (3.2)	2 (2.4)	1
Stroke, *n* (%)	5 (8.1)	12 (14.6)	0.343
Temporary, *n* (%)	3 (4.8)	9 (11.0)	0.310
Permanent, *n* (%)	2 (3.2)	3 (3.7)	1
Ischemic, *n* (%)	3 (4.8)	9 (11.0)	0.310
Hemorrhagic, n (%)	2 (3.2)	1 (1.2)	0.806
Spinal cord deﬁcit, *n* (%)	5 (8.1)	13 (15.9)	0.252
Temporary paraplegia, *n* (%)	2 (3.2)	3 (3.7)	1
Permanent paraplegia, *n* (%)	3 (4.8)	10 (12.2)	0.218
Renal failure necessitating dialysis, *n* (%)	3 (4.8)	9 (11.0)	0.310
Temporary, *n* (%)	3 (4.8)	7 (8.5)	0.594
Permeant, *n* (%)	0 (0.0)	2 (2.4)	0.604
Pulmonary complication, *n* (%)	15 (24.2)	26 (31.7)	0.422
Reoperation, *n* (%)	8 (12.9)	7 (8.5)	0.566
Gastrointestinal bleeding, *n* (%)	1 (1.6)	0 (0.0)	0.888
Gastroplegia, *n* (%)	4 (6.5)	3 (3.7)	0.704
Postoperative maximum ALT, U/L [median (IQR)]	120.02 (150.02)	159.28 (317.51)	0.37
Postoperative maximum AST, U/L [median (IQR)]	160.03 (231.89)	183.61 (322.24)	0.626
Postoperative maximum amylase, U/L [median (IQR)]	382.85 (436.88)	396.15 (467.68)	0.862

Values are *n* (%) or median [interquartile range].

ICU, intensive care unit; POD, post-operative day.

There were 12 early deaths (8.3%) in the whole cohort of patients, which included 3 early deaths in group A (4.8%) and 9 early deaths in group B (11.0%). One patient died intraoperatively due to cardiac arrest during the induction of general anesthesia, and this was considered an anesthetic allergy. Although the early mortality and incidence of stroke, spinal cord deﬁcits, renal failure necessitating dialysis were lower in group A than in group B, there was no significant difference between the two groups. However, the incidence of adverse events in group A was significantly lower than that in group B (9.7% vs. 25.6%, *P* = 0.027). The length of intensive care unit (ICU) stay and hospital stay were similar between the two groups. The incidence of reoperation was also similar between the two groups. All of these reoperation cases were caused by excessive bleeding postoperatively, and all of these patients needed re-exploration for hemostasis. Besides, there was no significant difference in the incidences of gastrointestinal complications and pulmonary complication between two groups (Table 3).

### Risk factors analysis

All of the preoperative and intraoperative characteristics were introduced into a single variable logistic regression model in which the dependent variable was defined as the presence of adverse events. Subsequently, eight variables were filtered out based on a *P*-value <0.1 and then were included in the stepwise multivariable logistic regression. Finally, five variables, including age >50 years, emergency surgery, Ccr < 90 ml/min/1.73 m^2^, the operating time and the previous FET implantation, were introduced into the risk model. Three variables, including age >50 years, a Ccr < 90 ml/min/1.73 m^2^, and the operating time, were identified as significant risk factors for adverse events of thoracoabdominal aortic repair under normothermic iliac perfusion (Table 4).

**Table 4:** Logistic regression analysis for adverse event.

Variables	Univariate logistic regression			Multivariate logistic regression		
	OR	95% Confidence Interval	*P*-value	OR	95% Confidence Interval	*P*-value
Connective tissue disorder	2.15	5.02–0.92	0.076	–	–	–
Blood loss	1.60	1.11–1.01	0.027	–	–	–
Previous FET implantation	0.31	0.83–0.12	0.019	0.43	1.38–0.13	0.154
Emergency	10.00	57.86–1.73	0.010	5.95	58.65–0.60	0.127
Age ≥50 years	3.97	10.11–1.56	0.004	3.87	11.76–1.27	0.017
Packed red blood cells	1.28	1.50–1.09	0.003	–	–	–
Operating time	1.04	1.06–1.02	0	1.04	1.06–1.01	0
Ccr < 90 ml/min/1.73 m^2^	8.56	26.36–2.78	0	4.71	16.04–1.38	0.013

OR, odds ratio; Ccr, creatinine clearance rate; FET, frozen elephant trunk.

### Follow up

**Figure 2:**
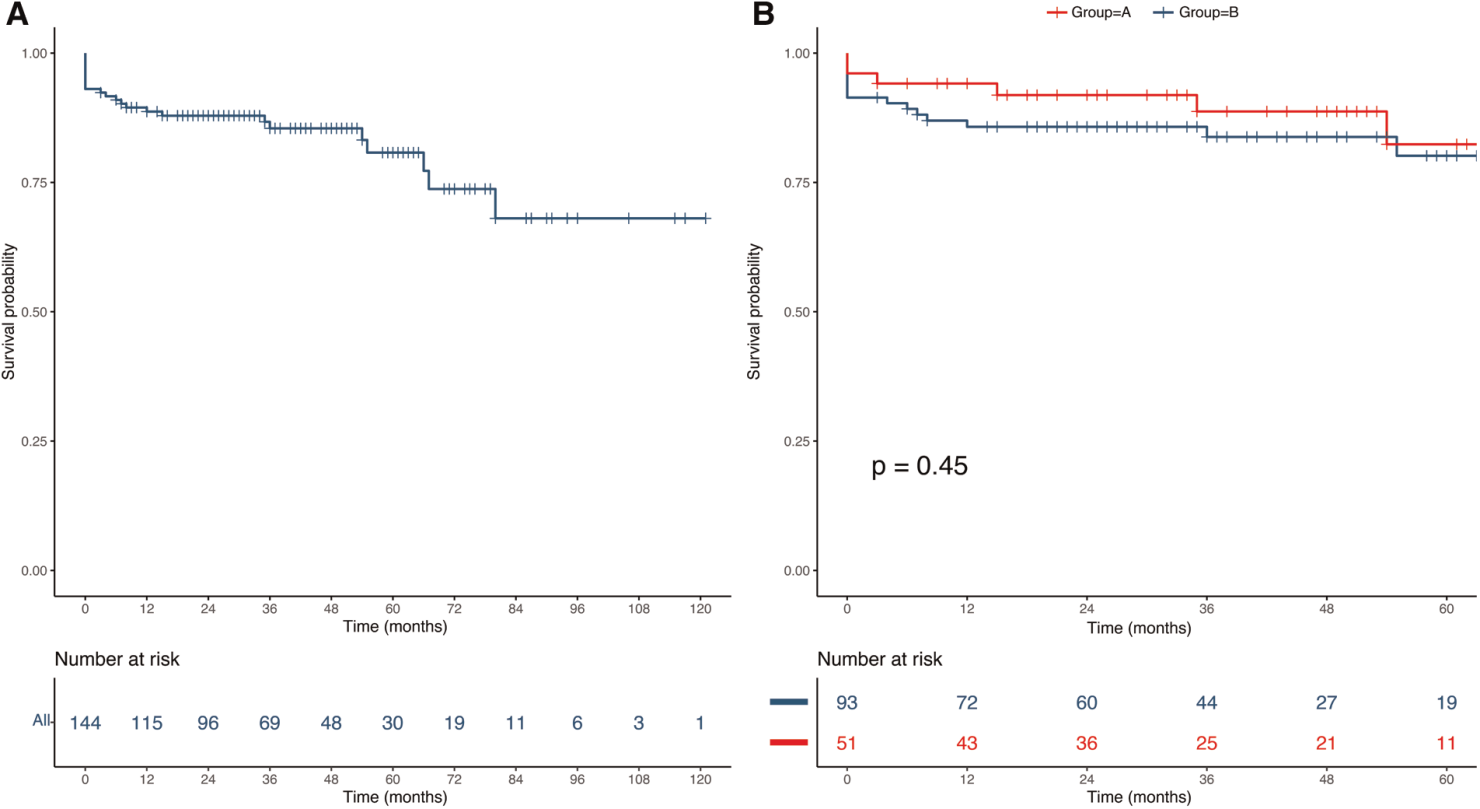
Kaplan–Meier curves of the estimated survival. (**A**) The overall mid-term survival curve for all of the patients; (**B**) The mid-term survival curve between group A and group B.

The median follow-up was 36 months (IQR: 21–54). Fourteen deaths occurred during follow-up, including five deaths in group A and nine deaths in group B. The overall estimated survival was 88.7% at 1 year, 80.8% at 5 years, and 68.1% at 7 years (Figure 2A). The log-rank test indicated that there was no significant difference in the postoperative survival rates between group A and group B (91.9% vs. 85.7 at 1 year, 82.4% vs. 80.1% at 5 years, *P*-value = 0.45) (Figure 2B). In addition, K-M curve was cut off at 60 months due to the high standard error (>0.1 in the two groups).

## Discussion

Normothermic iliac perfusion in thoracoabdominal aortic repairs has been proven to be the preferable strategy to reduce early operative death and complications compared with DHCA (1). Nevertheless, under this strategy, there is no blood supply and there is no protection of hypothermia to the distal aortic segments when the proximal aorta is anastomosed with the proximal end of the 4-branched graft. Hence, more rigorous techniques and more experience are needed to reduce the time needed for the anastomoses and the time of organ ischemia. Borst and colleagues first reported that the application of the elephant trunk technique in staged extensive aortic repair made aortic clamping safer and reduced the aortic anastomosis time (2). Scott et al. also indicated that acceptable short-term and long-term outcomes were obtained through the elephant trunk technique for extensive thoracic aortic aneurysms (9). Subsequently, as the aortic intervention techniques have been developed, a combination of surgical and interventional approaches named frozen elephant trunk (FET) were developed and were introduced into routine clinical practice, which provided additional value to the conventional elephant trunk by creating a landing zone for subsequent TA repair (3, 4, 10, 11). The FET technique can promote aortic remodeling in 99% of patients after aortic dissection and can achieve a 65% event-free survival at 8 years in patients with Marfan syndrome with acute type A dissection (12, 13). However, aortic reinterventions are common despite the closure of the proximal entry tear, which is probably due to the persistent increased pressure of the false lumen from distal communications (14).

Based on previous literature, the effect of the FET technique for TA repair involves three aspects (9, 15–17). First, anastomoses with FET are quicker and easier, which reduces the time of organ ischemia, and organ ischemia can lead to renal failure and spinal cord ischemia. Especially in the normothermic iliac perfusion strategies, the reduction of the time needed for proximal anastomoses should create greater benefits. Finally, FET eliminates the need for dissection near the distal arch and proximal thoracic aorta, which reduces injury to the esophagus and local nerves. In this study, we evaluated the role of FET in TA repair under a normothermic iliac perfusion strategy by comparing the clinical outcomes of patients with or without previous FET implantations.

In our cohort, we found that not only the time needed for proximal aortic anastomoses was significantly reduced, but also the operating time was significantly shorter in the group of patients with a previous FET implantation compared with the group of patients without a previous FET implantation. There was no difference in the rate of early death or complications between the two groups among total cohort. For further analysis, adverse event, as a composite end point, was found to be significantly fewer in group A than that in group B. Hence, according to the results obtained, we pointed out that previous FET implantation as a protective factor could improve the early outcomes in TA repair.

The variables of adverse events were evaluated as dependent variables when we performed the risk factor analysis, mainly because a small number of dependent variables would generate a biased result in the regression analysis. In the univariate analysis, the history of FET implantation was identified as a protective factor and was introduced into the multivariable regression model. However, it was not an independent risk factor for the composite adverse events in the multivariable analysis. On the one hand, the FET technique mainly simplifies the operation of proximal anastomosis. On the other hand, the clinical outcomes were also affected by factors such as age, the preoperative renal function and the presence of a connective tissue disorder. In addition, operating time was identified as a risk factor. Hence, according to the above analysis, we believe that frozen elephant trunk improves the early outcomes by reducing operating time in thoracoabdominal aortic repair with normothermic iliac perfusion.

Based on our experience, the primary advantage of FET in TA repair is the simplification of the proximal anastomosis, including the reduction of the proximal anastomosis time and the decrease of complications associated with the technique of the proximal dissection. Proximal anastomosis with FET al.so reduces the hemostasis time needed for the proximal anastomosis during surgery. Notably, significant thrombus around the FET from endoleak also complicates the proximal anastomosis, which is contrary to the benefit of FET. Extensive thrombus between the aortic wall and FET increase the difficulty of stent dissection and proximal aortic clamp time. Therefore, during the aortic clamp, it is necessary to prepare alternatives, including cardiopulmonary bypass, hypothermia, etc. Cooley et al. indicated that the risk of paraplegia increases with the duration of the aortic cross clamping as a sigmoid curve. When the clamping time is less than 30 min, the risk is less than 10% (18). In fact, although there were a few patients with severe endoleak in our cohort, all aortic clamp time were less than 40 min and no alternate regimen were used. Furthermore, the routine aortic cross-clamp on FET was considered safe. No complications due to clamping the frozen elephant trunk stent included endoleak, stent migration and deformation were observed after operation in our study.

Loschi et al. reported that the 5-year clinical success of TA repair was 79% in patients with a previous FET implantation. Scott et al. reported that the long-term survival after completing the second stage of repair was 70% at 5 years (9, 19). To investigate the effect of FET on the mid- and long-term outcomes in our cohort, a Kaplan–Meier analysis was performed. However, the results showed that there was no difference between the two groups regarding the late outcomes. In theory, the late reintervention rate of the aorta should be lower than in patients without previous FET implantation because most patients with previous FET implantation have an extensive aortic repair, including aortic arch and ascending aortic repair, after TA repair. Regrettably, we were unable to obtain more information about the deceased patients from the family members; we could not obtain any details regarding reinterventions during the follow-up, so these further analyses were not performed.

### Study limitations

There were several limitations to this study. First, this was a retrospective study from a single center. Normothermic iliac perfusion in TA repairs has not been widely introduced into other centers, and we are not sure if the results apply to other centers due to the various experiences of the team regarding TA repair. Moreover, the data extracted from the medical records are not comprehensive but included the intraoperative details, such as the visceral ischemic time and some complications, such as arrhythmia and late reinterventions. Nevertheless, this study is the first to quantitatively analyze the role of FET in TA repair, and this study provides statistical evidence rather than a theoretical understanding. In addition, this study introduces more details about a novel surgical strategy for thoracoabdominal aortic repair.

## Conclusion

The FET technique simplifies the operative technique of proximal anastomosis, significantly decrease the operating time and improve the early outcomes in TA repair with normothermic iliac perfusion. Moreover, operating time, age and preoperative renal insufficiency are identified as an independent risk factor for adverse events. Whereas FET technique does not provide a significant benefit with regard to late outcomes. Long-term follow-up and studies with larger sample sizes are necessary for further confirmation.

## Data Availability

The raw data supporting the conclusions of this article will be made available by the authors, without undue reservation.
